# Incidence of Fracture Hospitalization and Surgery in Women Increases Steadily During the Puerperal and Lactation Period: A Retrospective Register‐Based Cohort Study in Finland From 1999 to 2018

**DOI:** 10.1002/jbmr.4571

**Published:** 2022-06-03

**Authors:** Lauri Nyrhi, Ilari Kuitunen, Ville Ponkilainen, Tuomas T. Huttunen, Ville M. Mattila

**Affiliations:** ^1^ Department of Surgery Central Finland Hospital Nova Jyväskylä Finland; ^2^ Faculty of Medicine and Health Technology Tampere University Tampere Finland; ^3^ Department of Pediatrics Mikkeli Central Hospital Mikkeli Finland; ^4^ Institute of Clinical Medicine and Department of Pediatrics University of Eastern Finland Kuopio Finland; ^5^ Heart Center Tampere University Hospital Tampere Finland; ^6^ Department of Musculoskeletal Surgery Tampere University Hospital Tampere Finland

**Keywords:** INCIDENCE, PUERPERIUM, LACTATION, FRACTURES, SURGERY

## Abstract

This retrospective cohort study assesses the incidences of major fractures and surgery in women during the puerperium and the lactation period in Finland between January 1, 1999, and December 31, 2018. Using nationwide data from the Finnish Care Register for Health Care and the Finnish Medical Birth Register, all women aged between 15 and 49 years with a fracture hospitalization within 12 months of delivery between 1999 and 2018 were included. During the study period, a total of 3140 fractures after delivery and 152,800 fractures of the female normal population of similar age were hospitalized. The incidence rate after delivery increased from 219/100,000 person‐years during the first 4 months to 310 fractures/100,000 person‐years during the latter 8 months of the first year after delivery. Altogether, 29% (*n* = 904/3140) of these fractures were treated operatively. The most common fractures were ankle and distal radius fractures, which made up one‐third of all fractures. The incidence of pelvic fracture hospitalization was 15/100,000 person‐years at 4 months after delivery, with an operation rate of 22%. Over half of all fractures occurred between 6 and 12 months after delivery (mean 6.6 months). The incidence of fracture hospitalization after delivery increased steadily during the puerperium and the lactation periods but remained lower than in the general population (age‐adjusted incidence 554/100,000 person‐years) with an incidence rate ratio of 0.51. However, a higher proportion of pelvic fractures were observed in the first months after delivery. Surgical rates were in line with the general population. Fractures of the wrist and ankle made up most of the fractures. © 2022 The Authors. *Journal of Bone and Mineral Research* published by Wiley Periodicals LLC on behalf of American Society for Bone and Mineral Research (ASBMR).

## Introduction

Fracture incidence has long been known to follow different epidemiological patterns, depending on age and sex. For example, fractures in men resemble a bimodal parabolic trend with high rates in children, adolescents, and older adults and low rates in the working population. In women, a stable rate with a rapid increase after menopause is observed.^(^ ^1^, ^2^ ^)^ Overall, women suffer more fractures than men. This is especially apparent in older populations, where low‐energy osteoporotic fractures rise in incidence.^(^ ^3^ ^)^ In young adults aged 18 to 49 years, 80% of fractures are the result of high‐energy trauma, with the most common mechanisms being falls from height and motor vehicle collisions. Interestingly, when compared to adults >50 years, young adults present relatively high incidences of fractures of the hands and feet, but low incidences of traditional osteoporotic fractures. Moreover, men are also greatly overrepresented in this age group compared to women.^(^ ^4^ ^)^

Pregnancy and lactation are known stress factors for calcium metabolism in women. Despite little change in nutrition, a small number of women develop transient osteoporosis of pregnancy (TOP) in the third trimester of pregnancy.^(^ ^5^ ^)^ Case reports have previously been published describing late‐pregnancy, delivery‐related, and lactation period osteoporotic fractures.^(^ ^6^, ^7^, ^8^ ^)^ Although a high proportion of mothers express lower than normal vitamin D and estrogen levels, the pathophysiology behind TOP is not yet fully understood.^(^ ^9^, ^10^ ^)^ Although radiological findings normalize within a few weeks of delivery, the effects of pregnancy and lactation on calcium metabolism normalize only slowly after normal menstruation has resumed and lactation has ended.^(^ ^5^, ^9^ ^)^

A recent population‐based cohort study from Finland showed that the fracture incidence in pregnant women was smaller than in the general population.^(^ ^11^ ^)^ However, considering the late onset and lasting effects of TOP during pregnancy and lactation, we hypothesized that mothers suffer more fractures during their puerperal and lactation period than women in the general population. In this study, we analyzed all major fractures leading to hospitalization among mothers during their puerperal and lactation period and the normal population in Finland between 1999 and 2018 to provide information on the incidence rates of such fractures.

## Materials and Methods

Data for this nationwide retrospective register‐based cohort study were obtained from the Finnish Health and Social Data Permit Authority (FinData).^(^ ^12^ ^)^ We combined data from the Finnish Care Register for Health Care and the Medical Birth Register. The Finnish Care Register includes hospital inpatient data as well as data from day surgeries and specialized outpatient care containing both hospital clinic and emergency room visits. The coverage and accuracy of the register regarding diagnoses and discharges has been proven to be excellent, although information regarding patient comorbidities is lacking.^(^ ^13^, ^14^, ^15^ ^)^ The Medical Birth Register contains information on all pregnancies ending in delivery after gestational week 21 + 6 or fetal weight >500 g. The validity and coverage of the register is excellent and has been estimated to cover 100% of newborns in Finland.^(^ ^16^ ^)^

Our study period was from January 1, 1999, to December 31, 2018. Patients were selected from the Care Register using all fracture diagnoses coded with the 10th Finnish version of the International Classification of Diseases (ICD‐10)^(^ ^17^ ^)^ and all orthopedic fracture surgery codes from the Finnish version of the Nordic Medico‐Statistical Committee (NOMESCO) classification (Appendix S1).^(^ ^18^ ^)^ All female patients aged 15 to 49 years at the time of the injury, defined as fertile by the World Health Organization (WHO), were included in the study.^(^ ^19^ ^)^

**Fig. 1:**
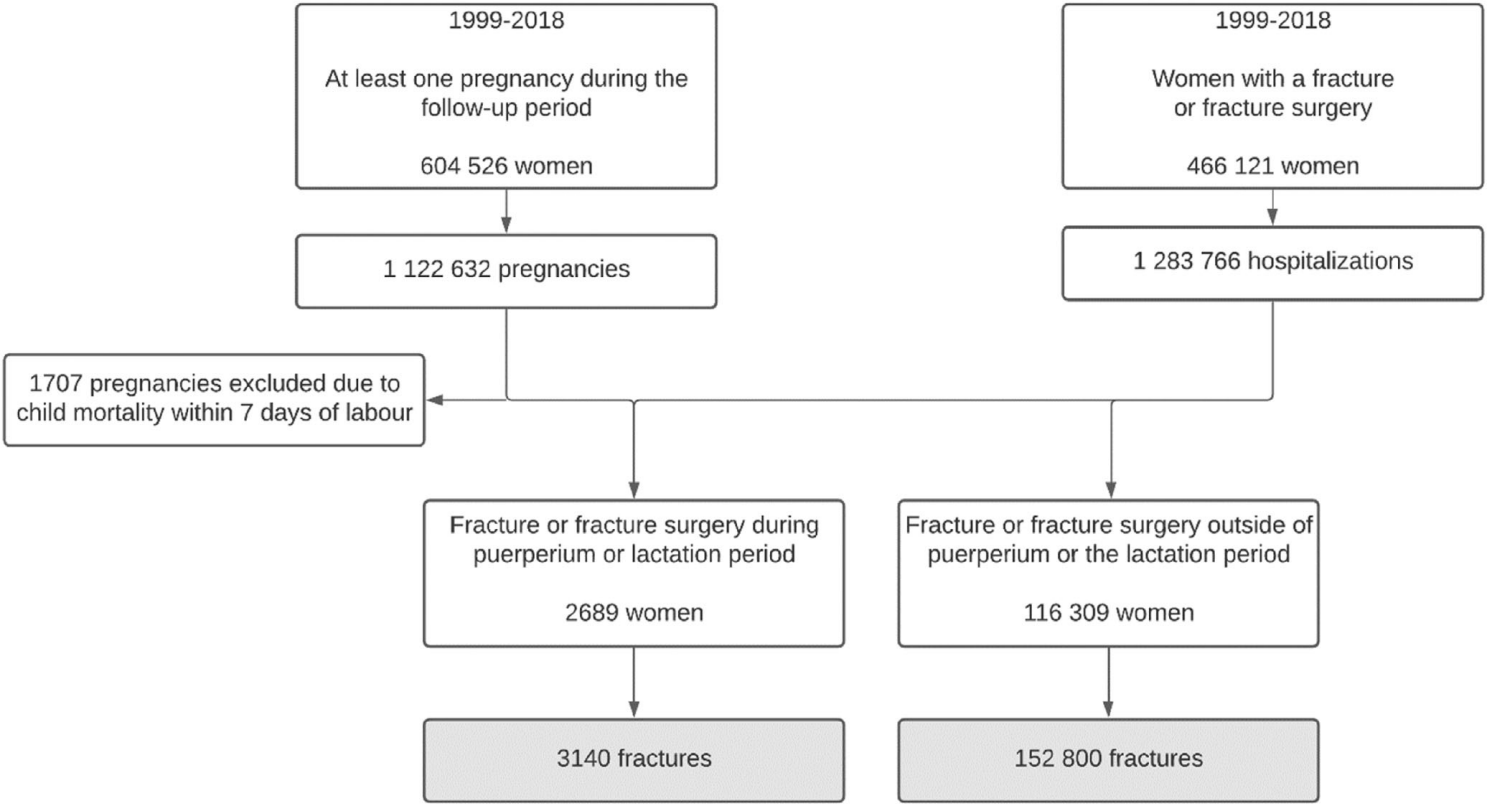
Flowchart of study cohort formation.

The registers were combined after the individuals were pseudonymized by FinData. The pseudonymization key was retained by FinData and none of the authors had access to the key. Additionally, all files were analyzed in the safe, remote‐controlled environment provided by FinData. Using information on date of birth and pregnancy duration from the Medical Birth Register, we were able to isolate incidents that occurred prior to or during pregnancy. In this study, the primary outcome was hospitalization with any of the fracture ICD‐10 or operation codes. Only the first hospitalization period per fracture was considered. The formation of the study cohort is described in Fig. 1.

This study was granted research permission from the Finnish Health and Social Data Permit Authority FinData, permission THL/1756/14.02.00/2020. According to Finnish research legislation and the Finnish National Board on Research Integrity appointed by the Ministry of Education and Culture, a review by a formal ethics committee is not required for the research of public and published data, registry and documentary data, and archive data.^(^ ^20^ ^)^ Our study was formatted according to the Strengthening the Reporting of Observational Studies in Epidemiology (STROBE) guidelines for observational studies (Appendix S2).^(^ ^21^ ^)^

### Statistical analysis

Yearly incidence rates (per 100,000 person‐years) were calculated for fracture hospitalization and fracture surgery. Separate calculations were made for follow‐up times of 4 and 12 months after delivery. This decision was based on the recommendation from the Finnish Institute for Health and Welfare that exclusive breastfeeding should be for a minimum of 4 months and complementary breastfeeding should be for up to 12 months.^(^ ^22^ ^)^ The Finnish recommendation differs from that of the WHO, as the WHO recommendation is for 6 months of exclusive breastfeeding and for the continuation of breastfeeding with complimentary nutrition up to 2 years of age.^(^ ^23^ ^)^ For the normal population of women of similar age, age‐adjusted incidence rates were calculated. Age‐adjustment was conducted by first calculating the crude incidence rates for each 1‐year age‐group of the normal population separately. These incidence rates were then weighted by multiplying the crude rate by the proportion of women in each age‐group of the postpartum population. These weighted rates were then summed to yield the final age‐standardized incidence rate. Incidence rate ratios (IRRs) and 95% confidence intervals (CIs) were calculated for incidence rates using Poisson regression. Regarding fracture onset, a Kaplan‐Meier survival analysis was performed for fracture patients to visualize the timing of the fracture relative to the number of months after delivery. Fractures were divided into anatomical subgroups and total incidences with 95% CIs were then calculated for the subgroups. All CIs were calculated using the Poisson Exact test. Fractures of the spinopelvic area and sites of traditional osteoporotic fractures were studied separately. For spinopelvic and osteoporotic fractures, we included the codes S22.0 (thoracic spine fracture), S32.0 (lumbar spine fracture), S32.1 (sacral fracture), S32.2 (coccygeal fracture), S32.3 (iliac fracture), S32.4 (acetabular fracture), S32.5 (pubic fracture), S32.7 (multiple pelvic or lumbar vertebrae fractures), S32.8 (other pelvic fracture), S33.4 (traumatic rupture of symphysis pubis), S72.0 (femoral neck fracture), S72.1 (pertrochanteric femoral fracture), S72.2 (subtrochanteric femoral fracture), S72.3 (femoral shaft fracture), S42.2 (proximal humeral fracture), and S52.5 (distal radius fracture). Fracture incidences with 95% CIs and operation percentages were calculated for these fractures. Fractures were considered surgically treated if fracture surgery was performed within 14 days of the first hospitalization. Patients with simultaneous multiple pelvic fractures were analyzed with code S32.7 regardless of primary coding. Statistical analyses were performed using R version 4.0.3 (R Foundation for Statistical Computing, Vienna, Austria; https://www.r-project.org/).

## Results

### All fractures

**Fig. 2:**
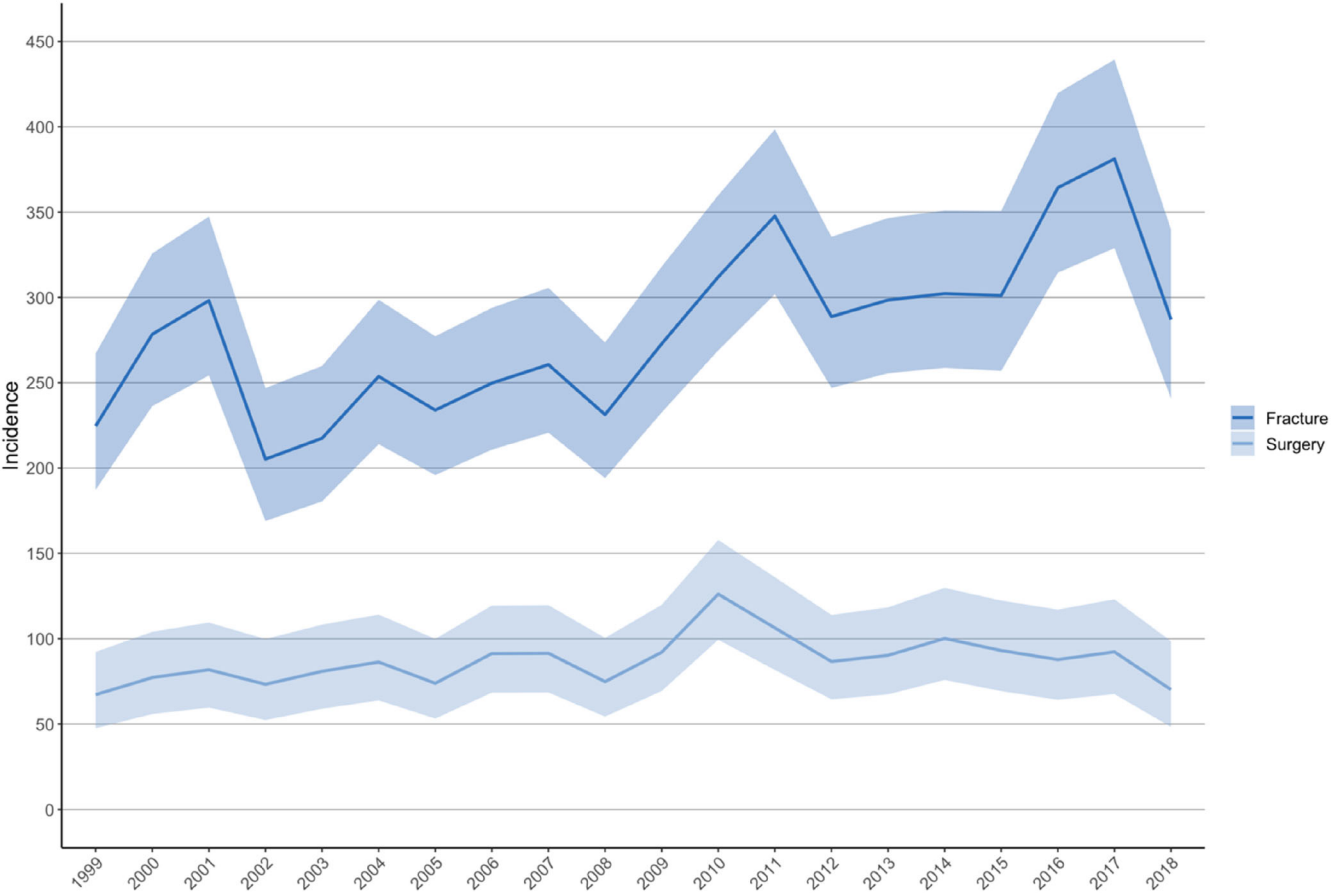
Yearly incidence (per 100,000 person‐years) of fracture hospitalization and surgery during the puerperal and lactation period in Finland between 1999 and 2018 and their 95% confidence intervals.

In our 20‐year study period, 2689 women were hospitalized with one or more fracture diagnoses during the first 12 months after delivery. In this population, a total of 3140 fractures were registered. The mean age ± standard deviation (SD) for all patients in postpartum women was 30 ± 5.7 years. For the normal population mean age was 33 ± 10.8 years. The 20‐year incidence rate for fracture hospitalization was 280/100,000 person‐years (95% CI, 270 to 290) and 87/100,000 person‐years for fracture surgery (95% CI, 61 to 71). The total incidence of fracture hospitalization during the first 4 months after delivery was 220/100,000 person‐years (95% CI, 205 to 235), rising to 310/100,000 person‐years during the next 8 months (95% CI, 297 to 323). In total, 29% of all fractures (*n* = 904/3140) required operative treatment. Annual incidence rates varied from 205 to 381/100,000 person‐years for fracture hospitalization and from 67 to 126/100,000 person‐years for fracture surgery (Fig. 2). For the normal population of women the respective 20‐year incidence for all fractures was 554 per 100,000 person‐years and 150 per 100,000 person‐years for fracture surgery. Women during their puerperal and lactation period suffered less fractures than the normal population with a total IRR of 0.51 (95% CI, 0.42 to 0.45; Table 1). 30% of all fractures were treated operatively in the normal population.

**Table 1:** IRRs of Fractures and Fracture Surgery of Postpartum Women and the Normal Population During the First Year After Delivery

Time period	Fracture IRR (95% CI)	Fracture surgery IRR (95% CI)	Fracture incidence postpartum (95% CI)	Fracture surgery incidence postpartum (95% CI)
0 to 12 months after delivery	0.51 (0.51–0.51)	0.5 (0.50–0.50)	279.7 (270.0–289.68)	87.47 (82.09–93.12)
0 to 4 months after delivery	0.4 (0.38–0.41)	0.4 (0.38–0.46)	219.66 (204.9–235.21)	65.47 (57.53–74.2)
4 to 12 months after delivery	0.56 (0.56–0.57)	0.55 (0.54–0.56)	309.72 (297.24–322.59)	87.92 (81.33–94.9)

Poisson exact test was used to calculate the IRRs, incidences, and their respective 95% confidence intervals.

IRR = incidence rate ratio.

**Fig. 3:**
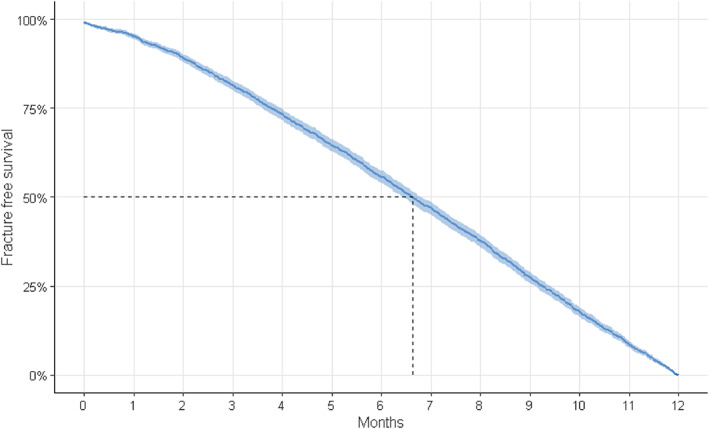
Temporal occurrence of fractures after pregnancy visualized in a Kaplan‐Meier survival graph.

The temporal occurrence of fractures within our 12‐month follow‐up period after delivery was fairly steady, with the mean onset of fractures occurring at 6.6 months after delivery. The incidence of fracture hospitalization showed an increase in the immediate postpartum period, before stabilizing and slowly increasing toward the end of our 12‐month follow‐up period. (Fig. 3).

### Anatomical distribution of fractures

During the first 4 months following delivery, the most common anatomical fracture location was the forearm (incidence 56/100,000 person‐years; Table 2) followed by the tibia and ankle (incidence 55/100,000 person‐years) and the hand (incidence 29/100,000 person‐years). Fractures of the distal radius accounted for 60% of forearm fractures (*n* = 125/210) and fractures of the distal tibia accounted for 64% of fibula tibia and ankle (*n* = 218/343) fractures. The incidence of pelvic fracture hospitalization was 15/100,000 person‐years, and the rate of surgical treatment was 22%.

**Table 2:** Incidences of Fractures and Fracture Surgery During the First 4 and the Latter 8 Months of the First Year After Delivery Divided by Anatomical Location

	Postpartum women					
	0–4 months after delivery		4–12 months after delivery		Normal population	
Anatomical location	Fracture incidence (95% CI)	Fracture surgery incidence (95% CI)	Fracture incidence (95% CI)	Fracture surgery incidence (95% CI)	Fracture incidence (95% CI)	Fracture surgery incidence (95% CI)
Head	11.22 (8.09–15.17)	0.27 (0.01–1.49)	21.11 (17.95–24.67)	0.13 (0–0.74)	41.22 (36.48–46.43)	0.4 (0.09–1.23)
Spine	12.56 (9.23–16.7)	1.87 (0.75–3.85)	15.23 (12.56–18.3)	0.8 (0.29–1.74)	27.94 (24.06–32.28)	4.09 (2.74–5.92)
Pelvis	14.7 (11.07–19.13)	3.21 (1.66–5.6)	9.22 (7.17–11.67)	1.34 (0.64–2.46)	12.53 (10.00–15.52)	1.99 (1.10–3.36)
Brachium	18.44 (14.35–23.34)	5.88 (3.68–8.9)	26.46 (22.9–30.41)	6.55 (4.84–8.66)	48.56 (43.40–54.17)	14.31 (11.64–17.43)
Forearm	56.39 (49.03–64.53)	9.09 (6.29–12.7)	78.3 (72.09–84.9)	17.9 (15–21.21)	120.91 (112.67–129.59)	24.27 (20.76–28.24)
Hand	28.86 (23.68–34.84)	10.42 (7.41–14.25)	46.36 (41.61–51.51)	18.04 (15.12–21.35)	84.3 (77.44–91.61)	27.96 (24.18–32.20)
Thigh	4.01 (2.24–6.61)	2.67 (1.28–4.91)	4.14 (2.81–5.88)	2.67 (1.63–4.13)	10.86 (8.50–13.68)	5.47 (3.88–7.52)
Tibia and ankle	54.78 (47.54–62.82)	29.66 (24.4–35.72)	75.36 (69.27–81.84)	36.08 (31.9–40.65)	150.98 (141.77–160.62)	65.04 (59.12–71.31)
Foot	12.03 (8.77–16.09)	6.15 (3.9–9.22)	21.91 (18.69–25.54)	8.42 (6.47–10.77)	33.72 (43.40–54.17)	15.16 (12.42–18.37)
Total	219.66 (204.9–235.21)	65.47 (57.53–74.2)	309.72 (297.24–322.59)	87.92 (81.33–94.9)	553.8 (535.99–572.05)	149.96 (141.02–159.34)

Incidences reported as fractures or operations per 100,000 person‐years. Poisson exact‐test was used to calculate the 95% confidence intervals in brackets.

Compared to fracture incidence at 4 months after delivery, 12‐month incidences increased in all anatomical locations, with fractures of the pelvis being the only group displaying higher fracture incidences during the first 4 months after delivery. Pelvic fracture hospitalization incidence reduced to a 12‐month incidence rate of 9/100,000 person‐years (95% CI, 7 to 12). The incidence of fracture surgery increased from 65/100,000 person‐years to 88/100,000 person‐years. Although the incidence of fracture surgery increased for most anatomical fracture locations, incidence decreased for fracture surgery of the head, spine, and pelvis.

For the normal population all fracture locations were more common compared to the postpartum population except for fractures of the pelvis, where fracture incidence (13/100,000 person‐years; 95% CI, 10 to 16) was similar to that during the first 4 months after delivery. The most common fracture sites were the tibia and ankle (incidence 151/100,000 person‐years) and the forearm (incidence 121/100,000 person‐years). The largest difference in incidence was seen in fractures of the thigh where the incidence of the normal population was 2.6‐fold when compared to postpartum women (incidence 11/100,000 person‐years; 95% CI, 9 to 14). Incidence of fracture surgery was also higher in the normal population in all fractures, but for those of the pelvis where incidences were similar both between the 4 months of surgery (3/100,000 person‐years; 95% CI, 2 to 6), the latter 8 months of the first year after delivery (1/100,000 person years; 95% CI, 1 to 2) and the normal population (2/100,000 person‐years; 95% CI, 1 to 3).

### Spinopelvic and typical osteoporotic fractures after pregnancy

A total of 852 spinopelvic and typical osteoporotic fractures were observed during the first 12 months after pregnancy with a total incidence of 76/100,000 person‐years (95% CI, 71 to 81) (Table 3). The 41% increase in incidence from 4 months to 12 months after delivery previously observed in total fracture hospitalization was not observed here, because the incidence in the first 4 months after delivery was 69/100,000 person‐years (95% CI, 61 to 78; *n* = 257), rising 16% during the latter 8 months to 80/100,000 person‐years (95% CI, 73 to 86). Incidence rates of fractures still remained below those of the normal population of 127/100,000 person‐years (95% CI, 104 to 159). The incidence of fracture surgery after delivery remained similar from 17/100,000 person‐years at 4 months (95% CI, 13 to 22; 25%) to 21/100,000 person‐years at 12 months (95% CI, 17 to 24; 26%). The overall surgical rate was comparable to that of the normal population (30%; incidence 36/100,000 person‐years; 95% CI, 25 to 55). Most fractures displayed a slight increase in incidence from the first 4 months to the latter 8 months. The most significant increase was seen in traditional osteoporotic fracture locations: the thoracic spine, the distal radius, the proximal humerus, and the proximal femur, but incidence rates still remained below those of the normal population. The incidence of fracture hospitalization due to the thoracic spine increased from 2.1/100,000 person‐years (95% CI, 0.9 to 4.2) to 3.6/100,000 person‐years (95% CI, 2.4 to 5.3). For fracture hospitalization of the proximal humerus and distal radius, the incidence increased from 6.4/100,000 person‐years (95% CI, 4.1 to 9.5) to 10/100,000 person‐years (95% CI, 8.1 to 13) and from 33/100,000 person‐years (95% CI, 28 to 40) to 45/100,000 person‐years (95% CI, 41 to 51), respectively. For multiple spinopelvic and comminuted fractures, the incidence rate at 4 months after delivery was higher at 3.2/100,000 person‐years (95% CI, 1.7 to 5.6). The incidence rate subsequently declined to 2.5/100,000 person‐years at 12 months after delivery (95% CI, 1.5 to 4.0), being the only fracture with higher incidence during the first 4 months after delivery compared to that of the normal population. For these fractures, however, the incidence of fracture surgery increased from 0.3 (95% CI, 0.0 to 1.5, 8%) at 4 months to 1.1 (95% CI, 0.5 to 2.1, 42%) at 12 months. All traumatic fractures of the symphysis pubis occurred during the first 4 months after delivery (*n* = 11/11) with an incidence of 2.9/100,000 person‐years (95% CI, 1.5 to 5.3), a surgical rate of 55% (*n* = 6/11), and mean fracture onset of 34 ± 42 days after delivery. Further, all traumatic fractures of the symphysis pubic occurred after vaginal delivery.

**Table 3:** Incidences of Fractures and Fracture Surgery and Surgical Rates of Spinopelvic and Typical Osteoporotic Fractures During the First 4 and the Latter 8 Months of the First Year After Delivery

	Postpartum women					
	0–4 months after delivery		4–12 months after delivery		Normal population	
Anatomical location	Fracture incidence (95% CI)	Fracture surgery incidence (95% CI, surgical rate)	Fracture incidence (95% CI)	Fracture surgery incidence (95% CI, surgical rate)	Fracture incidence (95% CI)	Fracture surgery incidence (95% CI, surgical rate)
Fracture of thoracic spine	2.14 (0.92–4.21)	0.27 (0.01–1.49, 12%)	3.61 (2.38–5.25)	0.13 (0–0.74, 4%)	6.43 (4.65–8.68)	1.26 (0.56–2.44, 19%)
Fracture of lumbar spine	5.88 (3.68–8.9)	1.6 (0.59–3.49, 27%)	5.21 (3.71–7.12)	0.67 (0.22–1.56, 13%)	10.49 (8.17–13.27)	2.61 (1.55–4.16, 24%)
Fracture of coccyx	3.74 (2.05–6.28)	0 (0–0.99, 0%)	3.74 (2.49–5.41)	0.13 (0–0.74, 4%)	3.92 (2.56–5.75)	0.2 (0.02–0.91, 6%)
Fracture of proximal humerus	6.41 (4.11–9.54)	1.34 (0.43–3.12, 21%)	10.29 (8.12–12.86)	2.94 (1.84–4.45, 29%)	13.61 (10.97–16.71)	3.76 (2.45–5.54, 28%)
Fracture of distal radius	33.67 (28.05–40.09)	7.22 (4.75–10.5, 21%)	45.43 (40.73–50.52)	13.36 (10.87–16.25, 29%)	71.57 (65.27–78.31)	19.7 (16.50–23.36, 29%)
Fracture of neck of the femur	1.6 (0.59–3.49)	1.6 (0.59–3.49, 100%)	1.07 (0.46–2.11)	0.53 (0.15–1.37, 50%)	2.78 (1.68–4.36)	1.56 (0.79–2.82, 61%)
Pertrochanteric fracture	0.27 (0.01–1.49)	0.27 (0.01–1.49, 100%)	0.53 (0.15–1.37)	0.27 (0.03–0.97, 50%)	0.87 (0.33–1.91)	0.45 (0.11–1.31, 54%)
Subtrochanteric fracture	0.27 (0.01–1.49)	0 (0–0.99, 0%)	0.27 (0.03–0.97)	0.13 (0–0.74, 50%)	0.72 (0.25–1.69)	0.43 (0.12–1.26, 57%)
Fracture of femoral shaft	1.07 (0.29–2.74)	0.8 (0.17–2.34, 75%)	1.6 (0.83–2.8)	1.07 (0.46–2.11, 67%)	3.25 (2.03–4.94)	2.26 (1.27–3.72, 71%)
Fracture of sacrum	2.14 (0.92–4.21)	0.27 (0.01–1.49, 12%)	1.6 (0.83–2.8)	0 (0–0.49, 0%)	2.86 (1.74–4.46)	0.67 (0.23–1.62, 21%)
Fracture of ilium	0.27 (0.01–1.49)	0 (0–0.99, 0%)	0.53 (0.15–1.37)	0 (0–0.49, 0%)	1 (0.41–2.08)	0.19 (0.04–0.88, 20%)
Fracture of acetabulum	1.87 (0.75–3.85)	1.07 (0.29–2.74, 57%)	0.27 (0.03–0.97)	0 (0–0.49, 0%)	1.53 (0.75–2.80)	0.61 (0.18–1.55, 37%)
Fracture of pubis	1.6 (0.59–3.49)	0 (0–0.99, 0%)	1.6 (0.83–2.8)	0.13 (0–0.74, 8%)	2.17 (1.22–3.60)	0.39 (0.10–1.20, 15%)
Other fracture of pelvis	1.6 (0.59–3.49)	0.53 (0.06–1.93, 33%)	1.2 (0.55–2.28)	0.13 (0–0.74, 11%)	2.12 (1.18–3.54)	0.35 (0.07–1.15, 14%)
Traumatic fracture of symphysis pubis	2.94 (1.47–5.26)	1.6 (0.59–3.49, 55%)	0 (0–0.49)	0 (0–0.49, 0%)	0.09 (0.01–0.39)	0.04 (0–0.31, 42%)
Multiple pelvic fractures	3.21 (1.66–5.6)	0.27 (0.01–1.49, 8%)	2.54 (1.53–3.96)	1.07 (0.46–2.11, 42%)	3.41 (2.17–5.12)	1.49 (0.74–2.72, 41%)
Total	68.68 (60.54–77.61)	16.84 (12.94–21.54, 25%)	79.5 (73.24–86.15)	20.58 (17.46–24.1, 26%)	127.43 (103.58–159.16)	36.09 (24.74–55.73, 30%)

Poisson exact test was used to calculate 95% confidence intervals for the incidences in parentheses. Rates of surgical treatment are shown as a percentage of the total count.

## Discussion

In our nationwide study, the incidence of most fractures during the first 12 months after delivery remained lower than in the normal population with a total incidence rate ratio of 0.51. Indeed, only the incidences of pelvic fractures during the first 4 months after delivery were similar to those in the general female population of the same age as also reported in previous studies.^(^ ^24^, ^25^ ^)^ We also found the total incidence of fractures leading to hospitalization during the first 12 months after delivery to be 280 fractures/100,000 person‐years. Of these, one‐fourth of the fractures required operative treatment. The incidence of fracture hospitalization increased after the first 4 months after delivery, rising from 220/100,000 person‐years during the first 4 months after delivery to 310/100,000 person‐years during the latter 8 months. The most common fractures occurred in the distal radius and the ankle, which made up almost one‐third of all fractures. Moreover, nearly half of all surgically treated fractures were fractures of the tibia or ankle. Indeed, in the first 4 months after delivery, only pelvic fractures were more common, with traumatic ruptures of the symphysis pubis being distinctive to the puerperal period.

No data regarding the incidence of fractures during the puerperium and lactation periods have previously been published, and therefore we are unable to compare our figures to previously published ones. Farr and colleagues^(^ ^4^ ^)^ have previously reported the total fracture incidence of women aged 18 to 49 years in Minnesota, USA, to be 1135/100,000 person‐years, including both inpatient and outpatient data. Similar whole‐population settings have reported the whole‐population fracture incidences of women to be 1065 and 1413 fractures/100,000 person‐years.^(^ ^1^, ^26^ ^)^ Our fracture incidence values for the general population fall behind, but separate values for fractures are in line with previously reported values for the corresponding age group.^(^ ^4^, ^26^, ^27^, ^28^, ^29^, ^30^ ^)^ In a previous study, 25% of all fractures in women aged 18 to 49 years concerned fractures of the foot, whereas our study showed a major representation of ankle, distal radius, and proximal humerus in both patient groups.^(^ ^4^ ^)^

Lower incidences of hospitalized fractures in women during the puerperal and lactation period might be at least partly explained by lower risk‐taking behavior. Furthermore, lactating mothers are also encouraged to abstain from alcohol use, which is a known risk factor for traumatic events.^(^ ^31^ ^)^ Proportionally higher incidences of traditional osteoporotic fractures suggest the possibility of a clinical role for TOP, which is known to only affect a portion of parturients.^(^ ^9^ ^)^ Our finding that pelvic fractures occur in higher numbers during the puerperal and lactation period in the first 4 months after delivery support the theory of TOP, which is known to affect the femur, pelvis, and lumbar spine.^(^ ^5^ ^)^ Fracture surgery for pelvic and spine fractures was also seemingly more common during the first 4 months after delivery, which could support the hypothesis of an osteoporotic etiology. This, together with weight gain, comprising both lean and fatty mass gain, and transient hypocalcemia can lead to a higher susceptibility to trauma during the first months postpartum.^(^ ^9^, ^32^, ^33^ ^)^ In Finland, it is recommended that mothers slowly resume daily activities after 2 months postpartum. This slow return to normal life and employment is in line with our findings of increasing incidences toward 12 months after delivery, and that over half of all fractures occurred between 6 and 12 months after delivery.^(^ ^34^ ^)^ Together with previous knowledge of transient osteoporotic changes recovering slowly, a part of this increase in incidence could be attributed to the long‐term changes effects of lactation and calcium mobilization, together with the increased stress of physical activity after the first months after delivery.^(^ ^5^, ^9^ ^)^

The main strength of our study is the excellent national coverage of hospitalized fractures.^(^ ^13^, ^15^ ^)^ Combined with the excellent national coverage of the National Birth Register, we were able to create unique national data on hospitalized fractures during the lactation and puerperal period after delivery, with excellent external validity.^(^ ^35^ ^)^ A secondary strength of our study is the long follow‐up time of 20 years. As a limitation of the study, the Care Register only includes hospitalized fractures. Thus, non‐operatively treated minor fractures, such as fractures of the extremities, are primarily treated in a primary health care setting and are therefore possibly missing from the Care Register.

## Conclusion

Our results suggest that fracture incidences during the puerperium and lactation period remain lower than in the general population but increase steadily as mothers return to normal life and activities during the first year after delivery. However, pelvic fractures mainly occurred during the first 4 months after delivery, with incidence rates matching those of the general population. Operation rates remained stable and relatively low throughout the study period. We hope our results will provide an epidemiological cornerstone for future studies of fractures and osteoporosis in puerperal and lactating women.

## Author Contributions

**Lauri Nyrhi:** Conceptualization; data curation; formal analysis; investigation; methodology; software; validation; visualization; writing – original draft. **Ilari Kuitunen:** Conceptualization; data curation; formal analysis; investigation; methodology; project administration; supervision; validation; writing – review and editing. **Ville Ponkilainen:** Conceptualization; data curation; formal analysis; investigation; methodology; software; visualization; writing – review and editing. **Tuomas T. Huttunen:** Conceptualization; data curation; investigation; methodology; project administration; supervision; validation; writing – review and editing. **Ville M. Mattila:** Conceptualization; funding acquisition; investigation; methodology; project administration; resources; supervision; validation; writing – review and editing.

## Conflicts of Interest

None to declare.

## Supporting information


**Appendix S1** Supporting Information

**Appendix S2** Supporting Information

## Data Availability

Due to Finnish legislature, data from national registers is limited and not publicly available.
