# Supplementary material for: Incidence of Fracture Hospitalization and Surgery in Women Increases Steadily During the Puerperal and Lactation Period: A Retrospective Register‐Based Cohort Study in Finland From 1999 to 2018
Source: J Bone Miner Res. 2022 Jun 3;37(7):1279–86. doi: 10.1002/jbmr.4571 (PMC9543855; doi:10.1002/jbmr.4571)
Supplement: Supplementary file 1 — Appendix S1 Supporting Information [file JBMR-37-1279-s001.docx]

**Supplementary files**

Incidence of fracture hospitalization and surgery in women increases steadily during the puerperal and lactation period: a retrospective register-based cohort study in Finland from 1999 to 2018

Authors: Lauri Nyrhi­ M.D. ^1,2^, Ilari Kuitunen, M.D.-Ph.D. ^3,4^, Ville Ponkilainen M.D.-Ph.D. ^1^, Tuomas T Huttunen M.D.-Ph.D. ^5,6^, Ville M Mattila M.D.-Ph.D. ^2,6^

*^1^ Department of Surgery, Central Finland Hospital Nova, Finland*

*^2^ Department of Musculoskeletal Surgery, Faculty of Medicine and Health Technology, Tampere University, Finland*

*^3^ Department of Pediatrics, Mikkeli Central Hospital, Finland;*

*^4^ School of Medicine, University of Eastern Finland, Finland*

*^5^Heart Center, Tampere University Hospital, Tampere, Finland*

*^6^Faculty of Medicine and Health Technology, Tampere University, Tampere, Finland*

**Running title:** Incidence of fractures during pregnancy

**Category:** Original article

**Conflicts of Interest and Source of Funding:** None were declared

**Corresponding Author:**

Lauri Nyrhi, MD

Tampere University, Faculty of Medicine and Health Technology

Arvo Building, PB 100, 33014, Tampere, Finland

Email: [lauri.nyrhi@tuni.fi](mailto:lauri.nyrhi@tuni.fi)

**Supplementary file 1:** Diagnosis (ICD) and operation (NOMESCO) codes used in cohort collection

Diagnosis codes:

S02 S02.0 S02.00 S02.01 S02.1 S02.10 S02.11 S02.2 S02.20 S02.21 S02.3 S02.30 S02.31 S02.4 S02.40 S02.41 S02.47 S12 S12.0 S12.1 S12.2 S12.7 S12.8 S12.9 S22.0 S22.1 S22.2 S22.3 S22.4 S22.5 S22.8 S22.9 S32 S32.0 S32.1 S32.2 S32.3 S23.4 S23.5 S23.7 S23.8 S42 S42.0 S42.1 S42.2 S42.3 S42.4 S42.7 S42.8 S42.9 S52 S52.0 S52.1 S52.2 S52.3 S52.4 S52.5 S52.6 S52.7 S52.8 S52.9 S62 S62.1 S62.2 S62.3 S62.4 S62.5 S62.6 S62.7 S62.8 S72 S72.0 S72.1 S72.2 S72.3 S72.4 S72.7 S72.8 S72.9 S82 S82.0 S82.1 S82.2 S82.3 S82.4 S82.5 S82.6 S82.7 S82.8 S82.9 S92 S92.0 S92.1 S92.2 S92.3 S92.4 S92.5 S92.7 S92.9

Operation codes:

AAD00 AAD05 AAD10 AAD12 AAD15 AAD30 AAD40 AAD42 AAD99 ACC51 NAB92 NAC92 NAG40 NAG41 NAG42 NAG50 NAG51 NAG52 NAG53 NAG56 NAG57 NAG60 NAG61 NAG62 NAG63 NAG65 NAG66 NAG67 NAG70 NAG72 NAG99 NAH10 NAH20 NAH30 NAH60 NAJ00 NAJ10 NAJ12 NAJ20 NAJ22 NAJ30 NAJ32 NAJ99 NAK10 NAK30 NAK40 NAK99 NAT00 NAT10 NAT12 NAT14 NAT20 NAT50 NAT60 NAT99 NAU10 NAU20 NAU99 NBA20 NBA30 NBB10 NBB15 NBB20 NBC20 NBC30 NBC40 NBE10 NBE15 NBE20 NBE25 NBE30 NBE35 NBE40 NBE45 NBE70 NBE72 NBE99 NBF10 NBF15 NBF20 NBF25 NBF30 NBF40 NBF99 NBG00 NBG01 NBG10 NBG15 NBG30 NBG60 NBG99 NBH10 NBH20 NBH30 NBH32 NBH97 NBH98 NBJ41 NBJ42 NBJ43 NBJ50 NBJ52 NBJ53 NBJ60 NBJ62 NBJ64 NBJ70 NBJ84 NBJ86 NBJ91 NBJ92 NBJ93 NBK02 NBK03 NBK10 NBK20 NBK30 NBK68 NBK70 NBK76 NBK91 NBK92 NBK93 NBK99 NBL00 NBL02 NBL04 NBL05 NBL22 NBL30 NBL50 NBL68 NBL99 NBQ10 NBQ20 NBQ48 NBM10 NBM20 NBT00 NBT50 NBT60 NBT99 NBW00 NBW10 NBW99 NCA20 NCA30 NCB10 NCB20 NCC20 NCC30 NCC99 NCE10 NCE15 NCE20 NCE25 NCE30 NCE35 NCF10 NCF15 NCR20 NCF25 NCF30 NCF40 NCF99 NCG00 NCG30 NCH NCH10 NCH20 NCH30 NCH32 NCH98 NCH99 NCJ40 NCJ44 NCJ60 NCJ62 NCJ64 NCJ70 NCJ84 NCJ86 NCJ99 NCK10 NCK20 NCK30 NCK68 NCK70 NCK99 NCL20 NCL22 NCL32 NCL40 NCL50 NCL60 NCL62 NCL64 NCL66 NCL99 NCM10 NCM20 NCM99 NCP10 NCP30 NCQ10 NCQ20 NCQ48 NCS10 NCS20 NCS99 NCT00 NCT50 NCT60 NCT99 NCU00 NCU10 NCU20 NCU30 NCU99 NCW00 NCW10 NCW99 NDA20 NDA30 NDB10 NDB20 NDB58 NDB60 NDB68 NDB70 NDB78 NDB99 NDC20 NDC30 NDC40 NDC99 NDE10 NDE15 NDE20 NDE25 NDE40 NDE60 NDE62 NDE64 NDE68 NDE99 NDF10 NDF15 NDF20 NDF25 NDF30 NDF60 NDF68 NDF69 NDF99 NDG00 NDG05 NDG20 NDG30 NDG39 NDG60 NDG61 NDG62 NDG63 NDG70 NDG71 NDG74 NDG76 NDG77 NDG79 NDH10 NDH20 NDH30 NDH32 NDH60 NDH62 NDH70 NDH72 NDH99 NDJ40 NDJ42 NDJ60 NDJ62 NDJ64 NDJ70 NDJ84 NDJ86 NDJ99 NDK00 NDK10 NDK30 NDK68 NDK99 NDL20 NDL22 NDL30 NDL32 NDL34 NDL40 NDL42 NDL52 NDL54 NDL60 NDL62 NDL64 NDL66 NDL99 NDM10 NDM20 NDM40 NDM50 NDM99 NDP10 NDP12 NDP18 NDP30 NDP32 NDQ10 NDQ20 NDQ48 NDQ60 NDR20 NDR30 NDR99 NDS00 NDS10 NDS20 NDS64 NDS99 NDT00 NDT10 NDT20 NDT32 NDT34 NDT36 NDT38 NDT40 NDT50 NDT99 NDU00 NDU02 NDU10 NDU20 NDU30 NDU50 NDU99 NDW00 NDW10 NDW99 NEA20 NEG30 NEG34 NEH20 NEH99 NEJ40 NEJ50 NEJ60 NEJ70 NEJ86 NEK10 NEK20 NEK99 NEL10 NEQ10 NEQ48 NER20 NER30 NER50 NES10 NES20 NET50 NET99 NEU10 NEU20 NEU99 NEW00 NEW10 NEW99 NFA20 NFA30 NFB10 NFB20 NFB30 NFB40 NFB50 NFB60 NFB62 NFB99 NFC00 NFC20 NFC30 NFC40 NFC50 NFC99 NFE10 NFE15 NFF20 NFF25 NFG00 NFG30 NFH10 NFH20 NFH30 NFH32 NFH99 NFJ40 NFJ42 NFJ50 NFJ52 NFJ54 NFJ60 NFJ62 NFJ64 NFJ70 NFJ84 NFJ86 NFJ99 NFK10 NFK20 NFK30 NFK50 NFK60 NFK70 NFK76 NFK99 NFL20 NFL22 NFL30 NFL68 NFL99 NFM NFM10 NFM20 NFM99 NFQ10 NFQ20 NFQ48 NFR20 NFR30 NFR80 NFR99 NFS10 NFS20 NFS99 NFT00 NFT50 NFT60 NFT99 NFU00 NFU10 NFU20 NFU99 NFW00 NFW10 NFW99 NGA20 NGA30 NGB10 NGB20 NGB30 NGB40 NGB50 NGB60 NGB99 NGC00 NGC20 NGC30 NGC40 NGC60 NGC99 NGD00 NGD05 NGD10 NGD15 NGD20 NGD25 NGD30 NGD35 NGD50 NGD60 NGD76 NGE10 NGE12 NGE15 NGE20 NGE25 NGE30 NGE35 NGE40 NGE45 NGE50 NGE55 NGE60 NGE65 NGF00 NGF10 NGF20 NGF25 NGF30 NGF35 NGF40 NGG00 NGG30 NGG34 NGH NGH10 NGH20 NGH30 NGH32 NGH99 NGK40 NGJ45 NGJ50 NGJ60 NGJ62 NGJ64 NGJ70 NGJ84 NGJ86 NGJ99 NGK00 NGK05 NGK20 NGK30 NGK50 NGK60 NGK70 NGK76 NGK99 NGL20 NGL22 NGL30 NGL34 NGL38 NGL50 NGL58 NGL64 NGL66 NGL99 NGM10 NGM20 NGM99 NGP10 NGQ10 NGQ20 NGQ48 NGR20 NGR30 NGR40 NGR99 NGS10 NGS20 NGS99 NGT00 NGT50 NGT60 NGT99 NGU00 NGU10 NGU20 NGU30 NGU40 NGU99 NGW00 NGW10 NGW99 NHA20 NHA30 NHB10 NHB20 NHC20 NHC30 NHE10 NHE15 NHE20 NHE25 NHE80 NHF10 NHF15 NFF20 NHF25 NHF26 NHF30 NHF70 NHF80 NHG15 NHG20 NHG22 NHG24 NHG26 NHG70 NHG76 NHG80 NHG82 NHG88 NHH10 NHH32 NHH40 NHH80 NHH99 NHJ08 NHJ10 NHJ12 NHJ40 NHJ50 NHJ80 NHJ86 NHJ99 NHK10 NHK30 NHK40 NHK68 NHK99 NHL10 NHL14 NHL16 NHL20 NHL30 NHL50 NHL68 NHL99 NHM10 NHM20 NHM99 NHP99 NHQ10 NHQ20 NHQ30 NHQ40 NHQ48 NHQ60 NHR20 NHR30 NHR99 NHS10 NHS20 NHS99 NHT00 NHT50 NHT62 NHT99 NHU00 NHU10 NHU20 NHU99 NHW00 NHW10 NHW99

**Supplementary file 2:** STROBE Statement—Checklist of items that should be included in reports of ***cohort studies***

|  | Item No | Recommendation | Page No |
| --- | --- | --- | --- |
| **Title and abstract** | 1 | (*a*) Indicate the study’s design with a commonly used term in the title or the abstract | 2 |
|  |  | (*b*) Provide in the abstract an informative and balanced summary of what was done and what was found |  |
| Introduction | | | |
| Background/rationale | 2 | Explain the scientific background and rationale for the investigation being reported | 3 |
| Objectives | 3 | State specific objectives, including any prespecified hypotheses | 3 |
| Methods | | | |
| Study design | 4 | Present key elements of study design early in the paper | 4 |
| Setting | 5 | Describe the setting, locations, and relevant dates, including periods of recruitment, exposure, follow-up, and data collection | 4 |
| Participants | 6 | (*a*) Give the eligibility criteria, and the sources and methods of selection of participants. Describe methods of follow-up | 4 |
|  |  | (*b*) For matched studies, give matching criteria and number of exposed and unexposed |  |
| Variables | 7 | Clearly define all outcomes, exposures, predictors, potential confounders, and effect modifiers. Give diagnostic criteria, if applicable | 4 |
| Data sources/ measurement | 8* | For each variable of interest, give sources of data and details of methods of assessment (measurement). Describe comparability of assessment methods if there is more than one group | 5-6 |
| Bias | 9 | Describe any efforts to address potential sources of bias | 4-4 |
| Study size | 10 | Explain how the study size was arrived at | 4-5 |
| Quantitative variables | 11 | Explain how quantitative variables were handled in the analyses. If applicable, describe which groupings were chosen and why | 5-4 |
| Statistical methods | 12 | (*a*) Describe all statistical methods, including those used to control for confounding | 5-6 |
|  |  | (*b*) Describe any methods used to examine subgroups and interactions |  |
|  |  | (*c*) Explain how missing data were addressed |  |
|  |  | (*d*) If applicable, explain how loss to follow-up was addressed |  |
|  |  | (*e*) Describe any sensitivity analyses |  |
| Results | | |  |
| Participants | 13* | (a) Report numbers of individuals at each stage of study—eg numbers potentially eligible, examined for eligibility, confirmed eligible, included in the study, completing follow-up, and analysed | 4 |
|  |  | (b) Give reasons for non-participation at each stage |  |
|  |  | (c) Consider use of a flow diagram |  |
| Descriptive data | 14* | (a) Give characteristics of study participants (eg demographic, clinical, social) and information on exposures and potential confounders | 4 |
|  |  | (b) Indicate number of participants with missing data for each variable of interest |  |
|  |  | (c) Summarise follow-up time (eg, average and total amount) |  |
| Outcome data | 15* | Report numbers of outcome events or summary measures over time | 7 |

| Main results | 16 | (*a*) Give unadjusted estimates and, if applicable, confounder-adjusted estimates and their precision (eg, 95% confidence interval). Make clear which confounders were adjusted for and why they were included | 7-11 |
| --- | --- | --- | --- |
|  |  | (*b*) Report category boundaries when continuous variables were categorized |  |
|  |  | (*c*) If relevant, consider translating estimates of relative risk into absolute risk for a meaningful time period |  |
| Other analyses | 17 | Report other analyses done—eg analyses of subgroups and interactions, and sensitivity analyses | 7-11 |
| Discussion | | | |
| Key results | 18 | Summarise key results with reference to study objectives |  |
| Limitations | 19 | Discuss limitations of the study, taking into account sources of potential bias or imprecision. Discuss both direction and magnitude of any potential bias | 12-14 |
| Interpretation | 20 | Give a cautious overall interpretation of results considering objectives, limitations, multiplicity of analyses, results from similar studies, and other relevant evidence | 13-14 |
| Generalisability | 21 | Discuss the generalisability (external validity) of the study results | 14 |
| Other information | | | |
| Funding | 22 | Give the source of funding and the role of the funders for the present study and, if applicable, for the original study on which the present article is based | 19 |

*Give information separately for exposed and unexposed groups.

**Note:** An Explanation and Elaboration article discusses each checklist item and gives methodological background and published examples of transparent reporting. The STROBE checklist is best used in conjunction with this article (freely available on the Web sites of PLoS Medicine at http://www.plosmedicine.org/, Annals of Internal Medicine at http://www.annals.org/, and Epidemiology at http://www.epidem.com/). Information on the STROBE Initiative is available at http://www.strobe-statement.org.
